# Design and testing of Hepatitis Delta Ribozymes for suppression of Chikungunya virus infection in cell cultures

**DOI:** 10.18103/mra.v12i8.5762

**Published:** 2024-08-31

**Authors:** Mark E. Fraser, Cheryl Kucharski, Zoe Loh, Erin Hanahoe, Malcolm J. Fraser

**Affiliations:** 1 Department of Pulmonology, Critical Care, Sleep, and Occupational Medicine, Indiana University School of Medicine, Indianapolis, IN 46202; 2 Department of Biological Sciences, University of Notre Dame, Notre Dame, IN 46556; 3 Department of Pathology, Duke University School of Medicine, Durham, NC 27710; 4 Moderna, 200 Technology Square, Cambridge MA 02139

**Keywords:** Chikungunya, CHIKV, Hepatitis Delta, Ribozyme, Antiviral

## Abstract

Chikungunya virus is an emerging pathogen with widespread distribution in regions of Africa, India, and Asia that threatens to spread into temperate climates following the introduction of its major vector, *Aedes albopictus*. Recent cases have been documented in Europe, the Caribbean, and the Americas. Chikungunya virus causes a disease frequently misdiagnosed as Dengue fever, with potentially life-threatening symptoms that can result in long term debilitating arthritis. There have been ongoing investigations of possible therapeutic interventions for both acute and chronic symptoms, but to date none have proven effective in reducing the severity or lasting effects of this disease. Recently, a promising vaccine candidate has received accelerated approval, indicating the importance of remedies to this emerging worldwide health threat. Nonetheless, therapeutic interventions for Chikungunya and other mosquito borne virus diseases are urgently needed yet remain elusive. The increasing risk of spread from endemic regions via human travel and commerce, coupled with the absence of a vaccine or approved therapeutic, puts a significant proportion of the world population at risk for this disease. In this report we explore the possibility of using Specific On/oFf Adapter Hepatitis Delta Virus Ribozymes as antivirals in cells infected with Chikungunya virus. The results we obtained suggest there could be some role in using these ribozyme molecules as antiviral therapies for not only Chikungunya virus, but potentially other viruses as well.

## Introduction:

Chikungunya virus (CHIKV) is a positive sense RNA virus (Family Alphaviridae) that is endemic to Asia, India, and parts of Africa, and is propagated by the bite of female Aedes (Ae.) mosquitoes, principally *Ae. aegypti* and *Ae. albopictus*. Blood-borne trans-mission is possible^1,2,3^ including vertical transmission^4,5^. The virus produces a disease that is characterized by abrupt onset of fever, myalgia, nausea, headache, fatigue, rashes, and debilitating arthralgia^6^. Other complications associated with CHIKV include myocarditis, hepatitis, ophthalmological, and neurological disorders^7^.

The CHIKV was first described in 1952 during an outbreak in southern Tanzania and has been identified in almost 40 countries to date. Recent epidemics include La Reunion Island in 2005–2006, with 255,000 cases among a total population of 750,000^8^ in India in 2006–2007, with 1.4 to 6.5 million estimated cases^9,10^, and the Philippines in 2013, with 180 cases. Cases of CHIKV have also been reported in parts of Europe as a result of infected individuals travelling from endemic regions^11,12,13^, and most recently in the Caribbean^14^. The rapid advancement of CHIKV worldwide corresponds to the spread of invasive *Ae. albopictus* and the advent of a CHIKV strain adapted to spread by this vector^15^.

A significant amount of work is underway to develop a vaccine for CHIKV. This includes strategies such as chimeric vaccines^16^, recombinant CHIKV vaccines^17^, adenovirus-based vaccines^18^, inactivated virus^19^, virus like particles^20^, and live attenuated vaccines^21,22^. Recently, a promising vaccine candidate entered clinical trials^23^, however, even with a viable vaccine there remain significant hurdles to effective prevention, containment, and control of any disease, especially in underdeveloped countries. There is a considerable amount of research devoted to the development of antivirals that may be useful as therapeutics for CHIKV disease^24^ including interferon^25^, deubiquinating enzyme inhibitors^26^, sphingosine kinase inhibitors^27^, radicol^28^ and defective viral genomes^29^.

The CHIKV is a small (60–70 nm-diameter) icosahedral, enveloped virus. The nucleocapsid contains a single stranded (+) sense genomic RNA of 11.8 kb that is organized 5’ cap-nsP1-nsP2-nsP3-nsP4-(Junction region)-C-E3-E2-6K-E1-poly (A)-3’. The non-structural proteins (nsP1, nsP2, nsP3 and nsP4) are encoded by the full-length genomic RNA and are responsible for replication of the viral genome^30^. A separate sub genomic RNA is produced during replication which encodes the structural proteins capsid, E3, E2, 6k, and E1^30^.

The two RNA species involved in the replication of CHIKV have proven to be excellent targets for RNA inhibition strategies. Antiviral strategies attempted thus far include RNA interference (RNAi) using small interfering RNAs (siRNA)^31^ or small hairpin RNAs (sh-RNA)^32^, each of which has demonstrated some success. While RNA interference can be an effective mechanism to suppress viral replication, this strategy requires targeting of highly conserved sequences of at least 21–23 nucleotides, which, in the case of CHIKV, limits the potential number of suitable target sites available. A single point mutation in an siRNA target site can result in decreased efficacy^33^, and the longer the target site, the more potential for these mutations. In addition, RNAi requires continuous synthesis of large amounts of ds-RNA in order to activate and maintain the RNAi machinery and effectively suppress viral replication^34^. Actively stimulating the RNAi response in a mouse model system has demonstrated a positive effect in overcoming CHIKV infection^35^.

Catalytic RNAs, ribozymes, can destroy viral RNAs in a targeted fashion leading to the elimination of viral genomic RNAs either entering the cell or produced during replication. Some of these catalytic RNAs are able to effectively regenerate, lessening the need for their continual production. In addition, therapeutic potential as delivered antivirals for some types of catalytic RNAs (hammerhead and hepatitis delta ribozymes, in particular) has been previously demonstrated^36,37,38^. In our lab we have been exploring various ribozymes for different applications against arboviruses^39,40,41,42,43^.

This report presents evidence that the Hepatitis Delta Virus Ribozyme (HDV-Rz) may have potential as a deliverable antiviral for the control of CHIKV, and potentially other vector borne diseases. HDV-Rzs are integral in HDV replication, generating monomeric genomes and antigenomes with the antisense version capable of acting in a trans fashion^44^. This ribozyme seems to be a suitable candidate anti-viral inactivation system for human cells^45^ and has previously demonstrated inhibition of gene expression in vivo^46,47,48^. In this study we explored the potential of a SOFA delta ribozyme design^49,50^ to suppress the production of infectious CHIKV in Vero cells by targeting conserved CHIKV sequences, and demonstrated that transfection of these in vitro expressed effector RNAs following infection of Vero cells can significantly reduce CHIKV infection in cell cultures.

## Materials and Methods:

### VIRUS:

Due to the unavailability of BSL3 facilities we used the CHIKV 181/25 vaccine strain for these studies, which was kindly provided by Dr. Scott Weaver, University of Texas Medical Branch, Galveston, TX). The virus was propagated in Vero cell cultures originally obtained from ATCC, and maintained in DMEM (Sigma-Aldrich, USA) supplemented with 10% FBS (Atlanta Biologicals) and 1% Non-essential amino acids (Gibco, USA) at 37° C with 5% CO_2_ and passaged every 4 days. Virus was maintained by infection of cells at an MOI (Multiplicity of Infection) of 2 in serum free DMEM for 2 hours with gradual shaking at 37° C. After 2 hours 7 ml of DMEM with 10% FBS was added and incubated at 37° C for 2 days, and supernatants harvested and stored at −80° C. The stock virus was titered using TCID_50_ as described below.

### DESIGN OF SOFA HDV-RZ EXPRESSION PLASMIDS:

**Figure 1.**
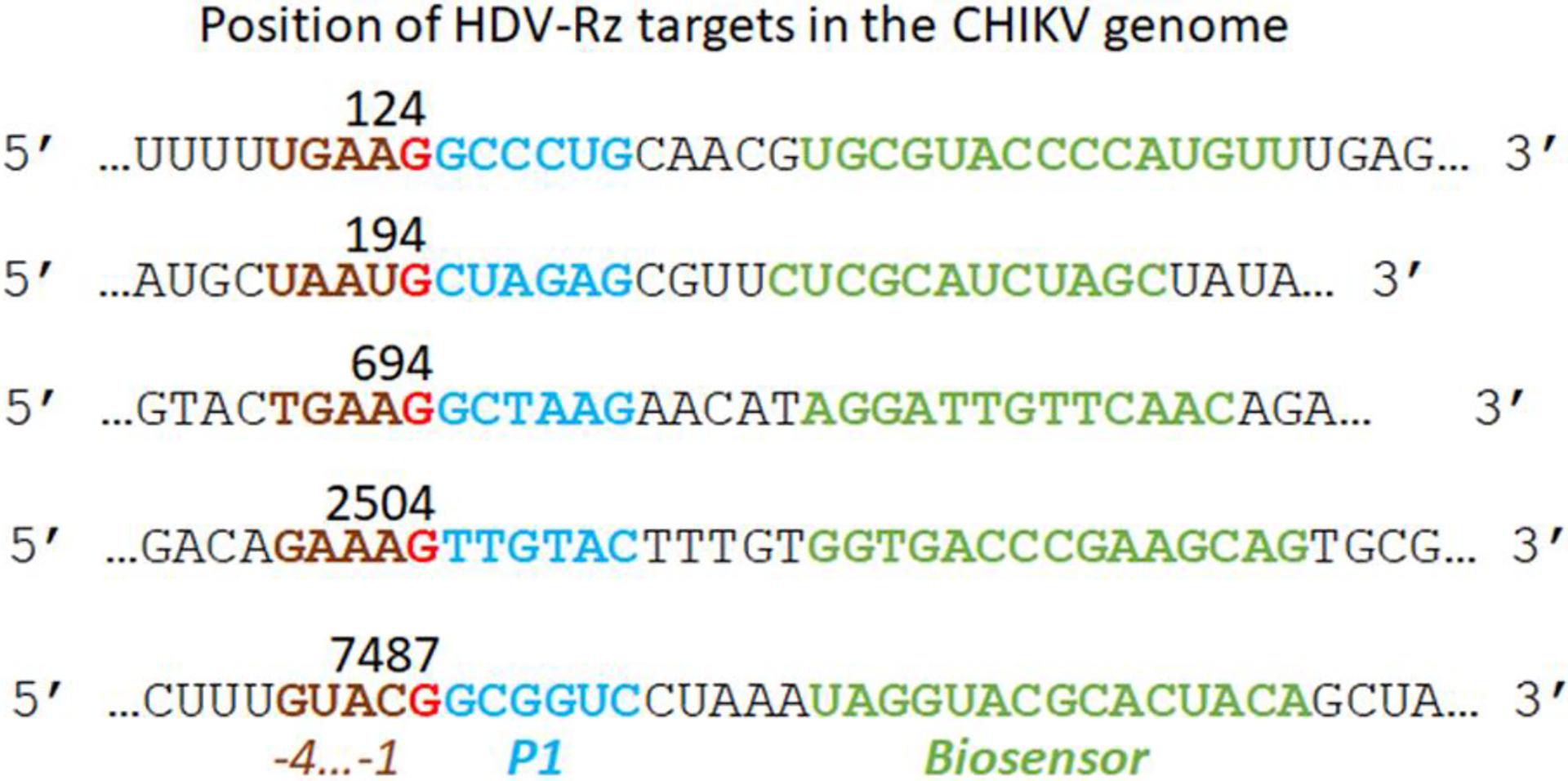
CHIKV genome sequences used to construct the corresponding antisense regions in the HDV-Rz (Table 1). The target cleavage site, G, is indicated in red.

The SOFA HDV-Rz we employed was based upon the original construct of Bergeron and Perreault^49^ and Levesque et al.^50^. Our antisense transacting SOFA HDV-Rzs modified from the published sequence with target sites selected from concensus conserved sequences having a −4YHRH-1 configuration, where Y=U or C, H=U,C, or A, and R=G or A^51^ (Fig. 1), from CLUSTALX alignments of 100 CHIKV strains available from the Gene Bank database. Target 124 does not conform to this consensus at position −3, 694 deviates at −4 and −3, and 2504 and 7487 at position −4. Target 194 conforms at all positions (Fig. 1).

**Figure 2.**
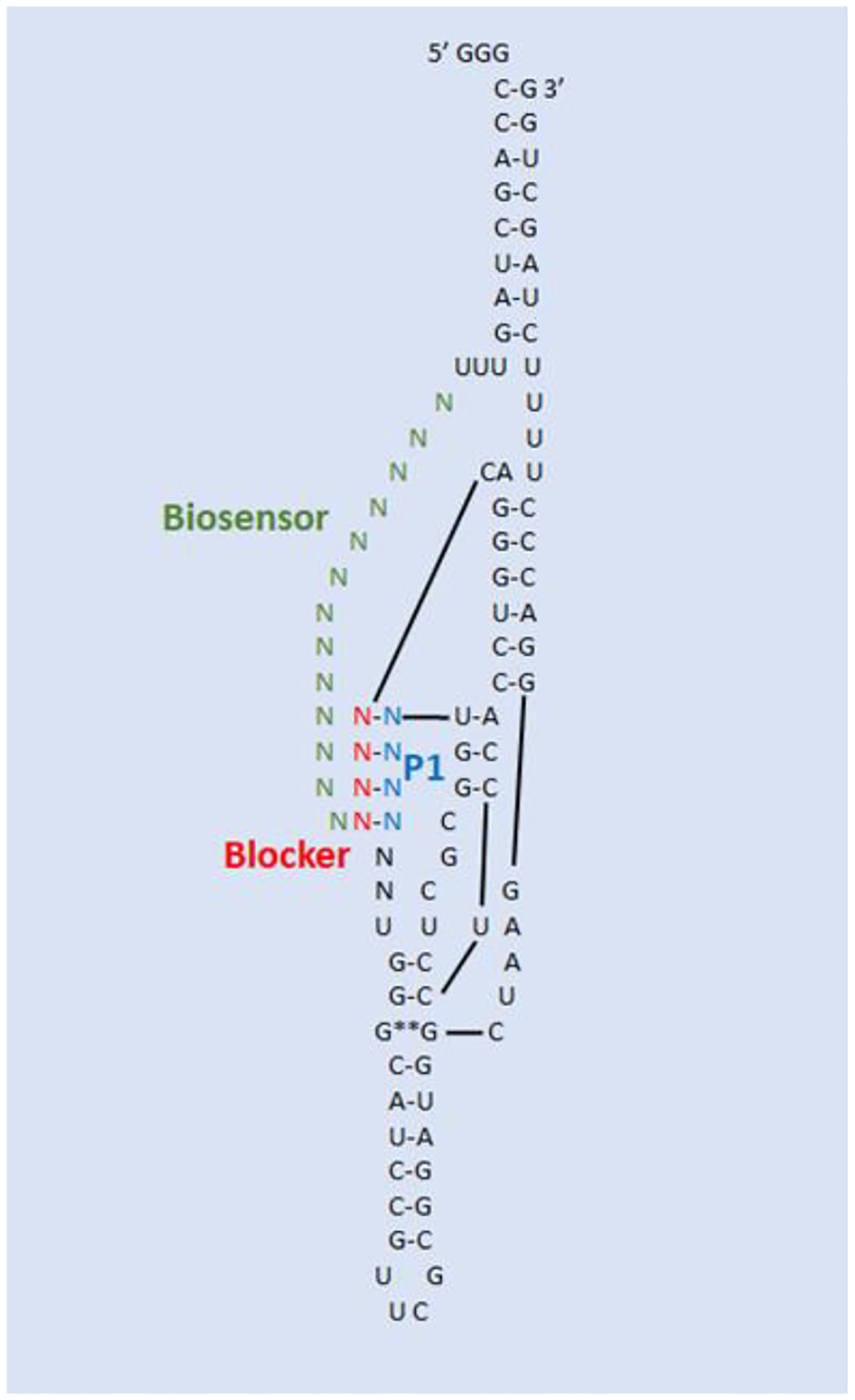
Schematic of antisense SOFA HDV-Rz. The relative position of the P1 targeting sequence is indicated in blue, its complementary Blocker sequence in red, and the CHIKV complementary Biosensor in green.

The relative locations of the different components of the SOFA HDV-Rz, Biosensor, P1 helix, and Blocker are more clearly explained in the results section and Figure 2.

Ribozyme expression plasmids were synthesized as T7-promoted expression cassettes. We also constructed inactive ribozymes having the same P1, biosensor, and blocker sequences as the active counterparts, but with a destabilized P1.1 helix through the replacement of GC nucleotides with A’s^52^, and an inactive active site by deletion of C75^53,54^.

### PREPARATION OF DELTA RIBOZYME RNA:

The SOFA HDV-Rz RNA was prepared by digestion of 1μg plasmid DNA with No-tI-HF (New England Biolabs) followed by purification with the E.Z.N.A. MicroElute DNA Clean-Up Kit (Omega Biotek). The DNA was eluted with 20 μl nuclease free water. The *in vitro* transcription reactions were performed using Invitrogen’s MEGAscript T7 Transcription kit according to the manufacturer’s directions, with 2 μL RNase Inhibitor, 6 μL digested and purified DNA, a final volume of 20 μL, and incubation overnight at 37° C. Following DNase treatment for 30 min, RNA was purified using phenol:chloroform:isoamyl alcohol (25:24:1; Sigma) extraction followed by ethanol precipitation with five 70 % ethanol washes and final suspension in nuclease free water. Purified RNA was used immediately for transfection.

### VIRUS INFECTION INHIBITION ASSAYS:

The effectiveness of SOFA HDV-Rz suppression of CHIKV infections was determined using a Caspase 3/7 assay kit (Promega) as previously described^43^. Vero cells were plated at 4 × 10^5^ cells per well in six well plates and infected with CHIKV at MOI 0.001, followed by incubation in DMEM (Sigma) with 2 % FBS for 4 hours. Owing to the robustness of CHIKV infections in this cell culture system^43^, infections were carried out at MOI 0.001 in order to observe inhibition of a progressing infection and to insure detection of differences in caspase levels before total cell mortality. The cells were then washed twice with serum free medium and 2 μg of each SOFA HDV ribozyme RNA, or nuclease free water (control) were transfected into one infected and one uninfected well each, using Lipofectamine 2000 (Invitrogen) following the manufacture’s recommendations. The transfection media was removed and replaced with 1.5 mL DMEM 2% FBS at 7.5 hpi and cells incubated at 37° C 5% CO_2_. After 48 hpi, 500 μL media was removed, mixed with 500 μL FBS, and stored at −80° C for later assay. The remaining media was removed from each well and 600 μL of a 1:1 ratio PBS pH 7.4 (GIBCO) and Caspase-Glo 3/7 Reagent (Promega) was added. The plate was incubated in the dark for 10 min with gentle rocking then an additional 5 min with no rocking. The 600 μL was then divided across three wells of a white walled 96-well plate and luminescence read with a LMaxII384 luminometer. The entire experiment, including RNA preparation and transfection of infected cells, was performed three times (on three separate dates) with each experiment testing the constructs/samples in triplicate, resulting in 9 total replicates for each experimental and 18 replicates for each control. Results were expressed as Relative Luciferase Units reported by the plate reader (RLU). Statistical analysis was performed within IBM SPSS via an ANOVA test followed by Tukey’s ad-hoc test to determine significance. Power was determined using a univariate analysis of variance within IBM SPSS with alpha set to 0.05 for the experiments.

### TCID_50_-IFA ANALYSIS OF CHIKUNGUNYA VIRUSES:

Viral supernatants were 10-fold serially diluted to 10^−8^ in 96 well plates and trypsinised Vero cells were added (2 × 10^4^/well) to the wells. After four days post infection (dpi), cells were fixed with Acetone: DPBS (3:1) and stained with (1:100) diluted CHIKV antibody (Catalog No: 3583 Virostat, USA). After incubation at 37° C for 40 minutes, stained cells were washed three times with 1x Dulbecco’s PBS (DPBS) and diluted (1:200) biotinylated anti-mouse antibody (RPN1001, GE Healthcare Life Sciences) was added to each well and again incubated at 37° C for 40 minutes. The cells were washed again three times to remove the unbound antibodies. Conjugated streptavidin-FITC (Catalog No: 434311, Invitrogen, USA) was added to the wells at a concentration of 1:150 and incubated for 10 minutes at 37° C. After washing two times with 1xDPBS and one time with distilled water, a drop of DABCO:glycerol (3.5 grams DABCO (Sigma- Aldrich,USA) + 10mL of 1xDPBS and 90 mL of glycerol) mix was added to each well and cytoplasmic fluorescence was observed using a Nikon Diaphot inverted fluorescent microscope. A total of six replicates were performed for both experimentals and controls. The number of positive green fluorescent wells were counted and the virus titers calculated according to Karber’s method^55^. The titer was expressed, as log_10_TCID_50_/ml. Data was log_10_ transformed for analysis as the data was logarithmic and not evenly distributed around the mean, and after log_10_ transformation the data was evenly distributed. Statistical analysis was performed on log_10_ transformed data within IBM SPSS via an ANOVA test followed by Tukey’s ad-hoc test to determine significance. Power was determined using a univariate analysis of variance within IBM SPSS with alpha set to 0.05 for the experiments.

## Results:

### STRUCTURE OF SOFA HDV RIBOZYMES:

The SOFA module of our HDV-Rzs acts as a switch to control the activity of the ribozyme by inhibiting the cleavage activity of the ribozyme with a blocker sequence that binds the P1 domain preventing formation of the catalytic configuration (Fig. 2). The blocker is adjacent to the biosensor sequence, which recognizes the target sequence on the substrate RNA. Once the biosensor binds the target, the blocker sequence is released from the P1 domain, permitting formation of the active conformation and cleavage of the target RNA^56^.

We synthesized T7 promoted expression cassettes of active G124, G194, G694, G2504 and G7487 SOFA HDV ribozymes, and inactive counterparts G124IN, G194IN, G694IN, G2504IN, and G7487IN. These inactive versions had the same P1, biosensor, and blocker sequences as the active counterparts, but the P1.1 catalytic helix replaced the GC nucleotides with A’s^52^, and the active site was inactivated by deletion of C75^53,54^. Lastly, the control HDV-R ribozyme retained an active P1.1 helix and active site sequence at C75, but replaced the P1, biosensor, and blocker sequences with randomly generated sequences not present in the CHIKV genome.

### ASSAY OF CASPASE 3 ACTIVITY IN CHIKV-INFECTED VERO CELLS AFTER LIPOFECTION WITH SOFA HDV-Rzs

The infection of cells by CHIKV results in an increase in caspase 3 levels as a result of virus induced apoptosis. In previous reports we have successfully used caspase 3 levels to measure relative levels of virus infection in cell cultures^43^. Caspase 3 was chosen as a benchmark for infectivity since viral infection and replication leads to caspase dependent apoptotic cell death, a primary defense response to limit viral replication^57^. In this analysis, Vero cells were first infected with CHIKV 181/25, and then transfected with *in vitro* transcribed SOFA HDV-Rz RNA. Following a 48 hour incubation period the culture supernatants were tested for caspase 3.

**Figure 3.**
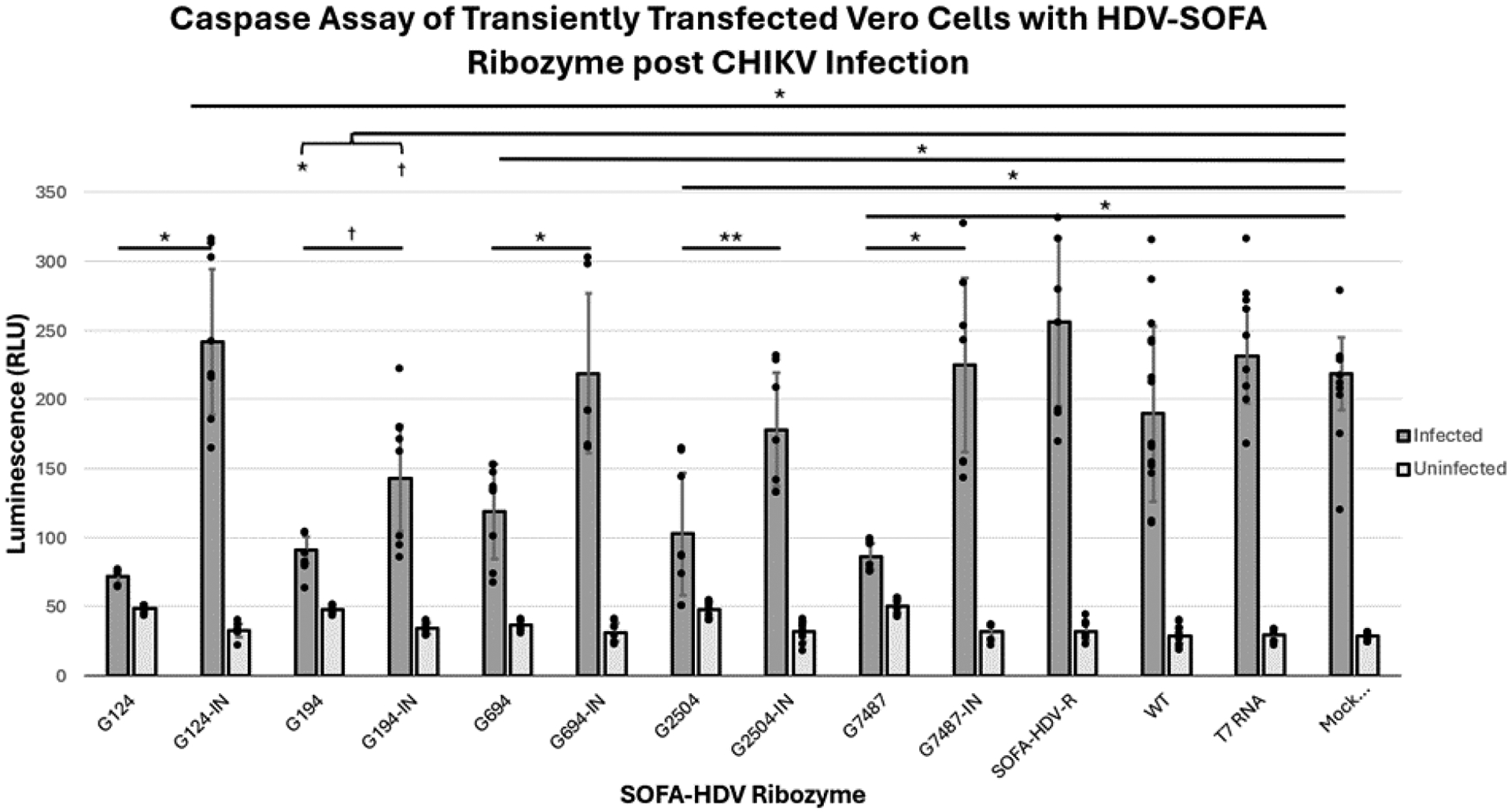
Caspase Activity of SOFA-HDV-Rz Transfected Vero Cells Challenged with CHIKV. Controls included untransfected VERO cells (WT), T7 Megascript RNA challenged VERO cells (T7 RNA), a mock reaction of transfection conditions without nucleic acid (Mock Transfection), and an active SOFA-HDV-Rz without biosensor (SOFA-HDV-R) as well as inactive ribozyme counterparts (-IN). Mean of data are plotted with error bars representing standard deviation, with all data points individually plotted for each replicate. *=p<0.001, **=p<0.01, †=p<0.05

Caspase 3 levels were not significantly different among all uninfected (active and inactive) SOFA HDV-Rzs when compared to each other and the controls, with p-values greater than 0.05 (Figure 3). Caspase activities of all but one of the infected samples were significantly higher compared to their uninfected controls (p<0.05). The sole exception was the infection of the G124 treatment which exhibited a slight but non-significant increase in caspase activity in the infected samples as compared with the uninfected samples (p>0.05) demonstrating greater suppressive activity than the other SOFA HDV Rzs (Table 2 and Figure 3).

All infected active SOFA HDV-Rzs, G124, G194, G649, G2504, and G7487 exhibited statistically significant reductions in caspase 3 levels compared with the infected controls: inactive corresponding ribozymes, Mock Transfection, SOFA HDV Ribozyme without biosensor (SOFA HDV-R), T7 random RNA (T7 RNA), and Wildtype (WT, un-treated Vero cells) (Table 2).

CHIKV infections treated with the five inactive control SOFA HDV-Rzs, G124IN, G194IN, G649IN, G2504IN, G7487IN, the SOFA HDV-R, T7 RNA, and Mock Transfection controls exhibited significantly increased CHIKV-induced apoptosis (as determined by the caspase 3 assay, Figure 3) compared to the WT control (p<0.05). The five inactive control SOFA HDV-Rzs, G124IN, G194IN, G649IN, G2504IN, and G7487IN were not significantly different in caspase activity from the SOFA HDV-R control, the T7 RNA control, or the Mock Transfection control (p-values all greater than 0.05). The SOFA HDV-R, T7 RNA, and Mock Transfection controls were not significantly different from each other (p-values > 0.05) (Table 2 and Figure 3). Combined, these data demonstrate that the introduction of RNA and/or the transfection process did induce some increase in caspase 3 activity within the cells in the infected samples, but the presence of the SOFA HDV-Rz themselves did not induce an increase in the caspase 3 activity above the increase due to the transfection/infection process.

The SOFA HDV-Rzs G194 and G194IN both significantly decrease CHIKV-induced apoptosis compared to the CHIKV infected Mock Transfection, SOFA HDV-R, T7 RNA, and the WT controls (p-value less than 0.001 for all). The SOFA HDV-R G194 showed significantly lower caspase 3 activity when compared to its inactive control (SOFA HDV-R G194IN) p-value < 0.05) (Table 2 and Figure 3). The reduction in caspase levels with the SOFA HDV-Rz G194IN suggests the predominant mechanism of inhibition for this ribozyme may be interference with CHIKV infection through binding at the target site.

### TCID_50_ ANALYSIS OF INFECTIOUS VIRUS PRODUCTION IN VERO CELLS TREATED WITH ANTI-CHIKV SOFA HDV-Rzs

The purpose of employing these SOFA HDV-Rzs is to inhibit infectious virus production thus lessening cellular infection. TCID_50_ assays are an effective means of measuring the production of infectious virus. Since many of the SOFA HDV-Rzs did not reduce the caspase levels of infected cells by at least 50%, we chose only G124 and G7487 to continue our analysis. Vero cells infected with CHIKV were transfected with each of the SOFA HDV-Rzs and allowed to incubate for 48 hours. Culture supernatants were harvested and used in a dilution titration assay to measure the relative effectiveness of the ribozymes in suppressing CHIKV production from infected cells.

**Figure 4.**
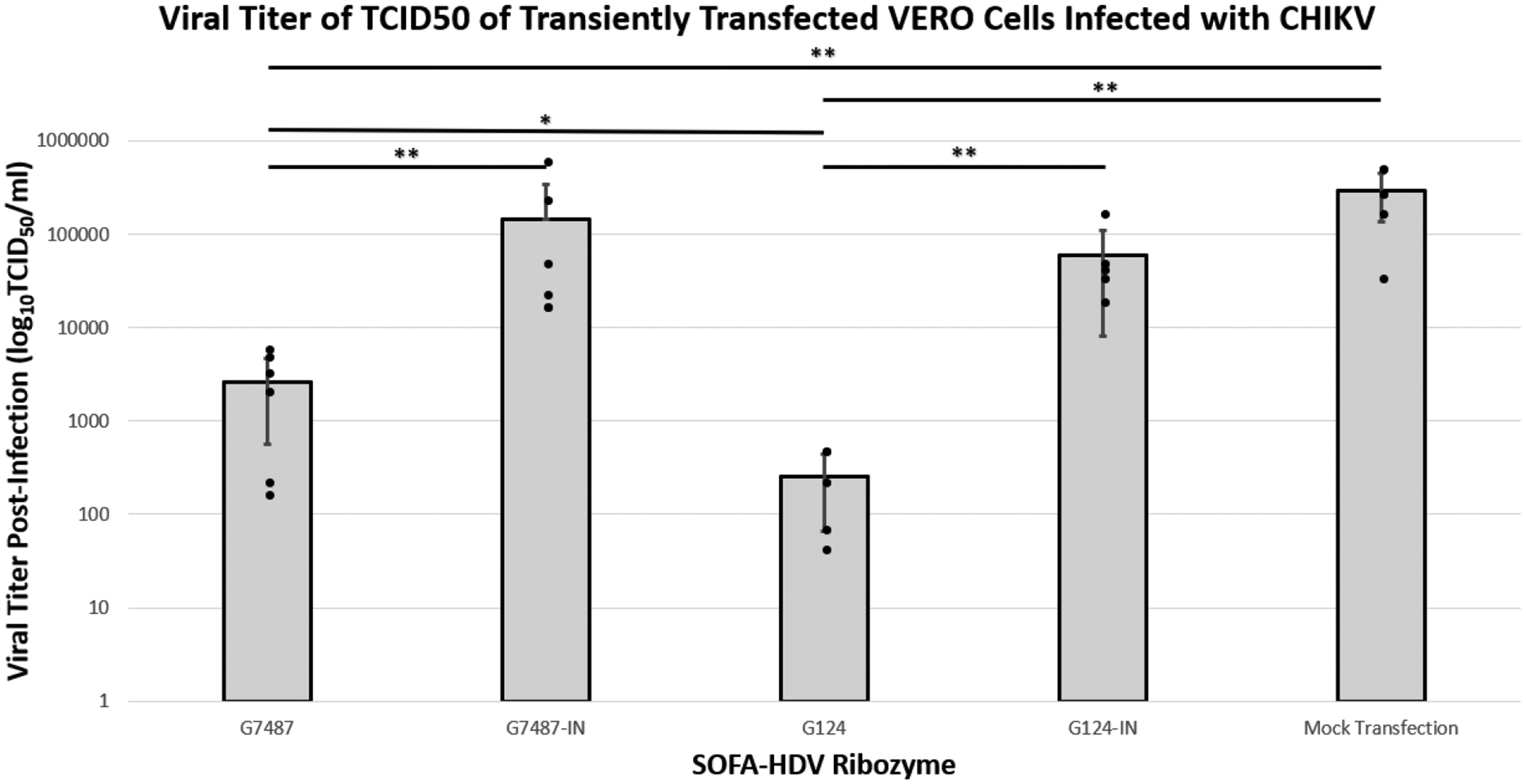
Viral Titer as Determined by TCID_50_ Assay of Transiently Transfected Vero Cells. G7487 and G124 yielded significantly reduced titers compared to the Mock Transfection control by and compared to their inactive controls (-IN). G124 was significantly reduced compared to G7487. Mean data are plotted with error bars representing standard deviation, with all data points individually plotted for each replicate. *=p<0.001, **=p<0.01

Both active SOFA HDV-Rzs G124 and G7487 displayed significant reductions in the viral titer from the Mock, with p-values less than 0.001 for both G7487 and G124 (Table 3 and Figure 4). In addition, the active SOFA HDV-Rzs significantly reduced the viral titers relative to their inactive controls. Ribozyme G124 showed a significant reduction from inactive ribozyme G124-IN with a p-value of less than 0.001 (Table 3 and Figure 4), and ribozyme G7487 showed a significant reduction from inactive ribozyme G7487-IN with a p-value of less than 0.001 (Table 3 and Figure 4). While SOFA HDV-Rzs G194, G694, and G2504 did exhibit some ribozyme activity based upon greater inhibition compared with their inactive counterparts, the level of activity was much lower than G124 and G7489. We therefore focused our analysis of virus productivity on the G124 and G7487 constructs.

## Discussion:

Chikungunya outbreaks have occurred in Africa, the Americas, Asia, Europe, the Caribbean, and islands of the Indian and Pacific Oceans, and represent an important economic burden in endemic areas. The most common symptoms of infection are fever and arthralgia, similar to those of Dengue fever. Other symptoms may include headache, myalgia, joint swelling/inflammation, or rash. No specific antiviral treatment is currently available for chikungunya; however, a number of therapeutic options are being investigated^24^. Among these are gene-inactivation approaches, including the use of antisense oligonucleotides, RNA interference (RNAi)^31^, and antiviral ribozymes^43^.

Our group has investigated antiviral hammerhead ribozyme transgenes for control of Dengue and Chikungunya viruses in mosquito and mammalian cell cultures^39,43^. While these types of ribozymes have proven effective in suppressing these viruses in cell culture, their translation to use as human antiviral therapeutics is less optimal than what may be achieved with HDV-Rz^50,57^.

The HDV was discovered in 1977 following the identification of a delta antigen (HDAg) in liver biopsies and sera from patients with a severe Hepatitis B. This antigen was then associated with a transmissible satellite virus that was dependent on the human Hepatitis B virus for packaging, release, and transmission^44^. Rolling circle replication of the negative-sense, genomic HDV RNA produces a complementary positive-sense, antigenomic RNA as the replication intermediate for synthesis of additional genomic RNA. Both genomic and antigenomic RNA concatemers are resolved into monomers by the HDV-Rz intrinsic ribozyme structure. The identification of HDV-like agents in a diversity of vertebrate and invertebrate species, and the structural similarity of the HDV ribozyme to cellular ribozymes suggests that HDV and its ribozyme likely evolved from the cellular transcriptome^44^.

In contrast with plant-derived ribozymes like hammerhead and hairpin, the HDV-Rz offers several unique properties as a potential human therapeutic tool including the natural adaptability to function in the presence of human proteins and physiological magnesium concentrations (1 mM Mg2+), and adopting a single conformation that is resistant to human nucleases leading to a long half-life. In addition, the use of liposome reagents is an efficient means of inducing cellular uptake of HDV-Rz^58^. Moreover, HDV-Rzs have demonstrated effectiveness in suppression of Hepatitis B *in vitro*^59^, HIV *in vitro*^60^, and Influenza infections in mice^52^.

In this study we employed a SOFA HD-Rz constructs based upon the original successful construct of Bergeron and Perreault^49^. The SOFA (Specific On/Off Adapter) module of this ribozyme acts as a switch to control the activity of the ribozyme by inhibiting the cleavage activity of the ribozyme with a blocker sequence that binds the P1 domain from forming the catalytic configuration. The blocker is adjacent to the biosensor sequence that recognizes the target sequence on the substrate RNA. Once the biosensor binds the target, the blocker sequence is released from the P1 domain, permitting formation of the active conformation and cleavage of the target^56^.

We chose caspase 3 as a measure of apoptosis induction to analyze the effectiveness of CHIKV infection suppression by these ribozymes. Since CHIKV replicates with very high efficiency in Vero cell cultures, the use of lower MOIs is preferred when examining the induction of cellular mechanisms such as apoptosis. Such mechanisms could not be analyzed effectively if the infection proceeded to complete destruction of the cells. This method of analysis has proven effective in our hands in previous analyses^43^.

We employed 5 CHIKV-specific biosensor sequences targeting different G residues within the CHIKV genome for cleavage (Table 1). Of these five, two exhibited the best suppressive activity upon lipofection of CHIKV infected Vero cells (Figures 3 and 4). Our controls (T7 RNA, SOFA HDV-R, and Mock Transfection) were not significantly different from each other, indicating the SOFA HDV-R lacking CHIKV targeting homologous sequences had no impact on viral induced apoptosis. These data confirm that the CHIKV targeting SOFA HDV-Rzs needs to interact with the genomic viral RNA in order to affect apoptosis and are not doing so in a non-specific manner.

While mutations in the P1 domain are not tolerated, mismatches in the biosensor region may be tolerated, depending on location relative to the P1 domain^61^. Thus, escape mutations may be somewhat less probable than would occur with RNAi or other ribozymes^33^. Certainly, the extent of homology maintenance for targeting and cleavage is considerably smaller than siRNA.

Depending upon which site is targeted, the SOFA HDV-Rz may inhibit CHIKV through simple biosensor RNA binding, or through the catalytic activity of the HDV-Rz. The observed statistically significant reductions in CHIKV activity relative to the catalytically impaired SOFA HDV-Rz controls suggests that these reductions are at least in part due to the catalytic activity of these SOFA HDV-Rzs. Targeting of G124 resulted in significant infectivity reductions as assessed by both the caspase and TCID_50_ assays, effectively reducing the viral titer by 3 logs TCID_50_. Similarly, targeting G7487 reduced the viral titer by 1.5 logs in TCID_50_ assays. These reductions, if realized in infected patients, could be sufficient to reduce clinical manifestation of disease.

In other systems, variability in effectiveness of HDV-Rzs is associated with several factors including substrate conformation, length and sequence of the biosensor, and cellular location of target viral genome during replication and translation^50^. These factors often cannot be reliably predicted^61^. However, there may be alternative sites in the genome that, while not necessarily consensus among all CHIKV strains, may prove more effective targets based upon these variables.

The use of many RNA species as therapeutic molecules requires engineering long term stability of these molecules. Some modifications can adversely influence their activity^62^. While the half-life of HDV-Rzs is superior to other RNA molecules, owing in part to its formation of a highly stable secondary structure^57^, this stability may be improved by chemical modifications as has been done for siRNA. However, these alterations can also decrease effectiveness, and in the case of SOFA HDV-Rzs, may not offer much advantage^63,64^.

Based on prior reports^64^ the infectiousness and productivity of the 181/25 strain in Vero cell cultures does not appear to be significantly different than virulent strains, However, there may still be some inapparent difference in the replication efficiency of the vaccine strain that influences these results. As a consequence, we can only say that the HDV-Rz approach shows some promise that needs to be replicated with virulent strains.

## Conclusion:

This report provides evidence that SOFA HDV-Rzs seem effective as antiviral agents against CHIKV in a cell culture infection situation. We demonstrated that post infection application of these ribozymes will effectively inhibit virus production, reducing the active infection significantly. These data provide evidence that such ribozymes may be useful as therapeutic tools in the treatment of CHIKV. In fact, such ribozymes have particular advantage in the treatment of human pathogens due to their relative stability and activity compared with hairpin and hammerhead ribozymes derived from plant sources. Even if the use of these SOFA HDV-Rzs such treatments were incapable of eliminating virus infection altogether, their application could contribute to a lessening of symptoms or duration of the disease. The simplicity of SOFA HDV-Rz construction allows the rapid examination of other potential target sites. In addition, the utilization of combinations of these ribozymes may prove even more effective. Based upon our relatively positive results we believe further investigation of the therapeutic potential of HDV-Rzs for viruses like CHIKV, Dengue, and Zika appears to be warranted.

## Figures and Tables

**Table 1.** Sequences correspond to locations in the schematic of the SOFA HDV-Rz, Figure 1.

SOFA-HDV-CHIKV Target Summary					
Name	Target	P1	Biosensor	Blocker	Target Region
G124	G	CAGGGC	AACAUGGGGUACGCA	CCUG	non-structural protein 1
G194	G	CUCUAG	GCUAGATGCGA	AGAG	non-structural protein 1
G694	G	CUUAGC	GUUGAACAUAAUCCU	UAAG	non-structural protein 1
G2504	G	GUACAA	CUGCUUCGGGUCACC	GUAC	non-structural protein 2
G7487	G	GACCGC	UGUAGUGCGUACCUA	GGUC	non-structural protein 4
Inactive	G	CCCCCC	CAGUUACUGU	GGGG	None

All sequences are 5’ to 3’, with the P1 and Biosensor sequences complementary to the CHIKV genome (Fig. 1), and the Blocker sequence complementary to the P1 sequence.

**Table 2.** Caspase activity of CHIKV infected Vero Cells treated post infection with SOFA- HDV-Rzs.

Construct	Mean (Caspase 3 RLU)	Standard Deviation (Caspase 3 RLU)	p-value compared to Mock	p-value compared to Inactive
*G124*	71.67	5.28	<0.001	<0.001
*G124-IN*	242.10	52.68	0.202	
*G194*	91.06	9.63	<0.001	0.012
*G194-IN*	132.36	46.31	<0.001	
*G694*	119.12	34.15	<0.001	<0.001
*G694-IN*	219.04	58.13	1.00	
*G2504*	102.92	44.25	<0.001	0.005
*G2504-IN*	178.08	41.12	0.796	
*G7487*	86.56	9.52	<0.001	<0.001
*G7487-IN*	225.08	63.12	0.849	
*SOFA-HDV-R*	256.27	60.44	0.136	
*WT*	189.67	63.38	0.024	
*T7 RNA*	234.73	34.30	0.419	
*Mock Transfection*	221.00	26.18		

Data presented were used to prepare the graph of Figure 3. Means and Standard Deviations are in caspase-3 Relative Luciferase Units (RLU). Statistics were performed in IBM SPSS via ANOVA with Tukey’s post-hoc test. Power for this experiment with alpha 0.05 was determined to be 1.000 utilizing IBM SPSS via Univariate Analysis of Variance.

**Table 3.** Viral Titers of CHIKV as Determined TCID_50_ Assay of Transiently Transfected Vero Cells.

Construct	Mean (TCID_50_/mL)	Standard Deviation (TCID_50_/mL)	p-value compared to Mock	p-value compared to Inactive
*G7487*	2632.55	2067.71	<0.001	<0.001
*G7487-IN*	146238.05	198934.72	0.24	
*G124*	250.80	183.96	<0.001	<0.001
*G124-IN*	58824.37	50740.36	0.15	
*Mock Transfection*	292477.82	155664.68		

Data used to prepare the graph of Figure 4. Mean and Standard Deviations are in TCID_50_/mL. Statistics were performed in IBM SPSS via ANOVA with Tukey’s post-hoc test. Power for this experiment with alpha 0.05 was determined to be 1.000 utilizing IBM SPSS via Univariate Analysis of Variance.
